# Multidisciplinary Therapeutic Approaches to Pancreatic Cancer According to the Resectability Status

**DOI:** 10.3390/jcm14041167

**Published:** 2025-02-11

**Authors:** Aurelio Mauro, Carlotta Faverio, Leonardo Brizzi, Stefano Mazza, Davide Scalvini, Daniele Alfieri, Alessandro Cappellini, Fabio Chicco, Carlo Ciccioli, Claudia Delogu, Marco Bardone, Anna Gallotti, Anna Pagani, Francesca Torello Viera, Andrea Anderloni

**Affiliations:** 1 Gastroenterology and Endoscopy Unit, IRCCS Foundation Policlinico San Matteo, 27100 Pavia, Italy; 2 Medical Oncology Unit, Fondazione IRCCS Policlinico San Matteo, 27100 Pavia, Italy; 3 Institute of Radiology, Fondazione IRCCS Policlinico San Matteo, 27100 Pavia, Italy; 4 Department of Internal Medicine and Medical Therapeutics, University of Pavia, 27100 Pavia, Italy; 5 Gastroenterology & Digestive Endoscopy Unit, AO Lodi, 26900 Lodi, Italy; 6 Section of Gastroenterology and Hepatology, PROMISE, University of Palermo, 90133 Palermo, Italy

**Keywords:** pancreatic ductal adenocarcinoma, EUS-guided intervention, biliary drainage, chemotherapy, neoadjuvant therapy, ERCP, staging

## Abstract

Pancreatic ductal adenocarcinoma (PDAC) is among the most lethal cancers, characterized by late diagnosis, rapid progression, and limited therapeutic options. Despite advancements, only 20% of patients are eligible for surgical resection at diagnosis, the sole curative treatment. Multidisciplinary evaluation is critical to optimize care, stratifying patients based on resectability into resectable, borderline resectable, locally advanced, and metastatic stages. Preoperative imaging, such as computed tomography (CT) and endoscopic ultrasound (EUS), remains central for staging, for vascular assessment, and tissue acquisition. Endoscopic and systemic approaches are pivotal for addressing complications like biliary obstruction and improving outcomes. Endoscopic retrograde cholangiopancreatography (ERCP) has been considered for years the gold standard for biliary drainage, although EUS-guided drainage is increasingly utilized due to its efficacy in both resectable and unresectable disease. Systemic therapies play a key role in neoadjuvant, adjuvant, and palliative settings, with ongoing trials exploring their impact on survival and resectability chance. This review highlights the evolving multidisciplinary approaches tailored to the disease stage, focusing on biliary drainage techniques, systemic therapies, and their integration into comprehensive care pathways for PDAC. The continuous refinement of these strategies offers incremental survival benefits and underscores the importance of personalized, multidisciplinary management.

## 1. Introduction

Pancreatic ductal adenocarcinoma (PDAC), specifically pancreatic ductal adenocarcinoma, represents one of the most lethal malignancies, with a 5-year survival rate of approximating 9% [1].

The high mortality rate can be attributed to the absence or non-specificity of symptoms in early stages, the difficulty to evaluate the pancreatic gland with first line diagnostic examinations, the lack of an adequate screening method and the quick progression of the disease, the poor sensitivity of first line examinations such as abdominal ultrasound to detect small pancreatic masses, and the absence of screening programs related to the sporadicity of the disease in the majority of cases [2].

Imaging techniques such as contrast-enhanced computed tomography (CT), magnetic resonance (MRI), and endoscopic ultrasound (EUS) provide sufficient data to evaluate the resectability of the lesion and its relationship with main vessels. Moreover, the latter expresses its potential in detecting small masses (<2 cm) and providing adequate samples of the mass with tissue acquisition (TA) [3]. Nevertheless, for preoperative staging, CT is the cornerstone imaging technique owing to its multiparametric evaluation.

Establishing an appropriate staging of PDAC is essential in order to guarantee the proper treatment that also has implication on patients’ overall survival [4]. In this setting a multidisciplinary evaluation with a gastroenterologist, surgeon, oncologist, and radiologist, especially for the most challenging cases, is essential to allocate patients to the best treatment. Currently, surgery is the only curative treatment but less than 20% of patients are deemed operable at diagnosis [5]. The majority of patients with PDAC are therefore candidates for active oncological treatment or palliation.

Different chemotherapy protocols are available for patients with PDAC and different trials are ongoing, especially in the setting of borderline resectable patients, with the intent of increasing survival, which in recent years has slight improved [6].

However, the treatment of PDAC is often only palliative and targeted to the treatment of jaundice or PDAC complications, such as cholangitis, chronic pain due to neuroplexus infiltration, and gastric outlet obstruction. In this setting, interventional endoscopy plays a pivotal role. Endoscopic retrograde cholangiopancreatography (ERCP) with a transpapillary stent remains the gold standard for the drainage of jaundice in cases of distal malignant biliary obstruction (DMBO) [7]. Nevertheless, in recent years, the progress of biliopancreatic endoscopy has led to new possibilities in the treatment of PDAC complications. In particular, EUS-guided biliary drainage (EUS-BD) has been expanding and has provided satisfactory data of efficacy and safety in this setting [8].

The complexity of this scenario necessitates specialized expertise and a joint force between various medical units to offer a multidisciplinary and high-level experience to the patient.

This comprehensive review aims to assess the practical approach to PDAC according to its stage, especially focused on the management of biliary drainage and systemic therapies.

## 2. Definition of Pancreatic Cancer Stages

PDAC requires precise preoperative imaging evaluation to ensure accurate staging and to guide therapeutic decisions.

Accurate preoperative imaging is essential for identifying patients at high risk of residual disease post-surgery, stratifying patients into four groups. First, resectable, which should be referred to surgery; second, borderline resectable which may benefit from neoadjuvant therapy; third, locally advanced disease, a situation without metastasis but with a local involvement that makes the lesion locally unresectable; fourth, metastatic disease that does not benefit from surgical resection [6].

Imaging in the stratification of patients must take the following characteristics into account: (1) localization, extent, and size; (2) vascular involvement; (3) nodal involvement; (4) metastatic disease.

These parameters are assessed by both the TNM staging system by American Joint committee on cancer (AJCC) and the NCNN criteria for resectability, shown in Table 1 and Table 2 [9].

### 2.1. Location, Extension, and Size

The location of the tumor within the pancreatic gland is defined by its relationship with the surrounding structures, in particular with the vascular axes. About 60–70% of PDAC occur in the pancreatic head, approximately 10–20% in the body of the pancreas, and 5–10% arise in the tail [1]. The dimension of PDAC defines the T staging and the CT scan has a pivotal role for lesions ≥ T2 especially for T4 lesions considering the radiological ability to evaluate arterial infiltration [4]. CT scan could have a lower sensitivity, especially if compared with EUS, for T1 lesions (<2 cm) [10]. The spread of the tumor strongly depends on its location and size. In addition to hematogenous and lymph node metastases, PDAC can spread via direct invasion or perineural spread. Direct spread involves structures adjacent to the tumor. A head tumor may involve the duodenum and stomach, a tail tumor may affect the spleen, colon, kidney and left adrenal gland [11].

Extension to adjacent organs is not in itself a contraindication to surgery, as long as the organs involved can be safely removed to ensure surgical radicality and negative margins (R0) [12].

### 2.2. Vascular Involvement

Evaluating vascular involvement is essential for determining the resectability of pancreatic tumors.

**Figure 1:**
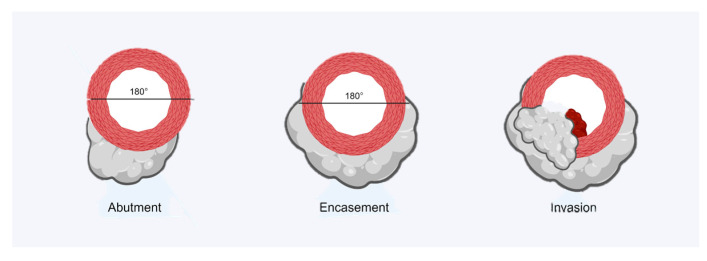
Visual representation of Abutment, Encasement, and Invasion of a vessel.

The degree of vascular involvement is defined by the anatomical relationship between tumor tissue and vessels, classifying the contact with the terms of abutment, encasement, and invasion [13] (Figure 1).

Abutment is defined as a circumferential contact of less than 180° between tumor and vessel; it is not considered a sign of vascular invasion. Encasement is defined when the contact between tumor and vessel exceeds 180°; this sign correlates with vascular invasion with a sensitivity of 84% and specificity of 98% [14]. Invasion is defined when contact with the tumor causes deformity of the vessel and often results in intravascular thrombosis, which is visible as a filling defect within the vessel during contrast medium scans [15].

#### 2.2.1. Invasion of Arterial Structures

To define full tumor resectability, there must be no contact between the tumor and the arterial vascular axis including the celiac axis (CA), SMA, and CHA.

On the other hand, PDAC of the pancreatic head or uncinate process is considered locally advanced and thus unresectable if the contact between tumor and SMA or CA is greater than 180°. If the tumor is located in the body-tail of the gland, it is considered unresectable if it has contact with the SMA or CA > 180° or if it involves the aorta.

Lesions are considered borderline resectable if they exhibit focal contact with the CHA, CA, or with an extension less than 180° with the SMA, or involve accessory arteries.

#### 2.2.2. Invasion of Venous Structures

For venous involvement, a tumor is considered locally advanced if it extensively involves the superior mesenteric vein (SMV) or the portal vein (PV) hampering surgical venous reconstruction.

On the other hand, patients are deemed operable if there is no venous involvement or if the tumor contact with the SMV or PV is ≤180°, whereas if the contact is >180° but surgical vascular reconstruction is allowable, it is defined as borderline resectable.

Additionally, involvement of the inferior vena cava (IVC) is also classified as borderline resectable if reconstruction is feasible [9].

### 2.3. Nodal Involvement

Characteristics of suspicious lymph nodes include a short-axis diameter exceeding 1 cm, a rounded shape, nodal clustering, loss of a fatty hilum, and cystic appearance. However, such loco-regional findings do not contraindicate surgery.

The detection of pathological lymph nodes in distant abdominal or extra-abdominal locations is considered indicative of metastatic disease and precludes surgical intervention [16].

### 2.4. Metastatic Disease

The presence of distant metastases establishes the unresectability of the tumor. Organs most frequently involved in metastases are the liver, peritoneum, and lungs.

Metastases from PDAC to parenchymatous organs often show the same imaging features as the primary.

As previously noted, CT can fail to detect small hepatic metastases. In these cases, MRI can aid in liver nodules characterization [17,18]. EUS could also detect small metastases in the left liver not evidenced by cross-sectional images [19]; however, to date, there are no standardized protocol to study liver parenchyma at EUS.

## 3. How to Stage Pancreatic Cancer?

### 3.1. Imaging Examinations

Multidetector CT (MDCT) is considered the gold standard for evaluating patients with suspected pancreatic masses. The sensitivity of MDCT for the diagnosis of PDAC rage between 70 and 100%, with a specificity of 70–100% and lower success rates (~67%) for smaller masses (≤1.5 cm) [20,21,22,23].

PDAC, if large, could be recognizable already on basal CT scan as a mass that modifies the normal lobulation of the gland, often hypodense due to a central necrotic [24,25]. The characterization of the lesion is based on the pancreatic phase, which usually appears as a hypovascular mass and often causes upstream dilation of the pancreatic duct [26,27].

**Figure 2:**
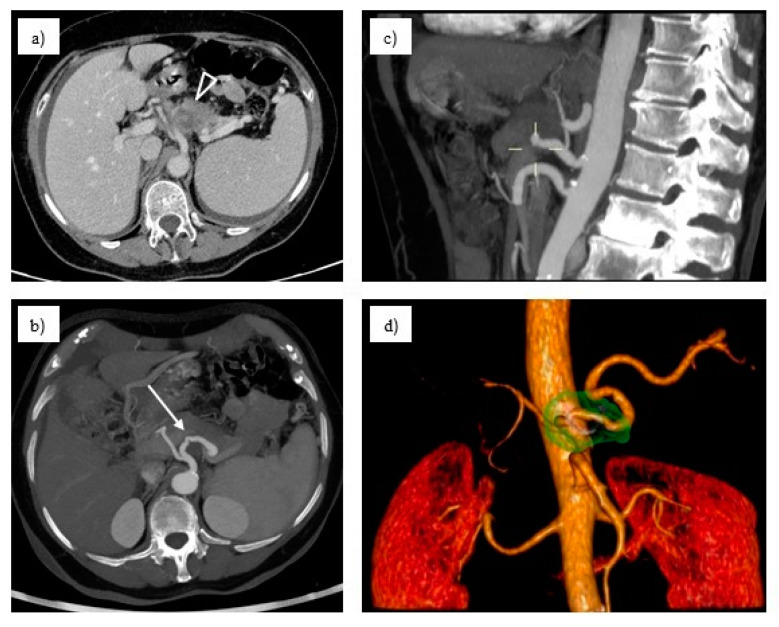
Imaging findings of pancreatic body adenocarcinoma. (**a**) Axial contrast-enhanced CT scan in the portal venous phase demonstrating hypovascular pathological tissue (arrowhead) encasing the proximal splenic artery. (**b**) Arterial-phase CT scan with oblique plane MIP reconstruction highlighting the irregular narrowing of the celiac trunk and splenic artery (white arrow), which is encased by hypovascular tumor tissue arising from the pancreatic body. (**c**) Arterial-phase CT scan with sagittal MIP reconstruction showing hypovascular tumor tissue completely surrounding the splenic artery, originating from the pancreatic body. (**d**) Three-dimensional volume rendering illustrating the spatial relationship between the tumor (highlighted in green) and the splenic artery, which is entirely encased by the neoplastic mass.

The pure arterial, venous, and late phases allow for the evaluation of tumor margins, the involvement of vascular structures, anatomic variants, and distant metastases. Vascular 3D reconstruction with CT-scan images allows for accurate evaluations of PDAC infiltration of the adjacent vascular structures and appropriate definition of the resectability status of the lesion (Figure 2) [4]. Moreover, a CT scan has the ability to detect non-regional nodal metastasis that place the patient in a not-operable status [28].

Magnetic Resonance Imaging (MRI) is not routinely applied in the detection, characterization, and staging of PDAC. However, it can be helpful in the detection of hepatic metastases and in isoattenuating lesions on CT scans due to its higher resolution in soft tissue [29].

PDAC often presents as a hypointense mass in T1-weighted sequences, with an intermediate signal in T2-weighted sequences, and restricted diffusion on DWI imaging, hypovascular after contrast [26,30]. Moreover, the use of contrast-enhanced ultrasonography (CEUS) can be performed alongside a standard US, injecting intravenously a sulfur-hexafluoride contrast medium (Sonovue, Bracco, Milan, Italy), which helps to improve the characterization of pancreatic masses.

### 3.2. Endoscopic Ultrasound

EUS has the ability to explore all the pancreatic gland and its relationship with adjacent organs and vessels.

**Figure 3:**
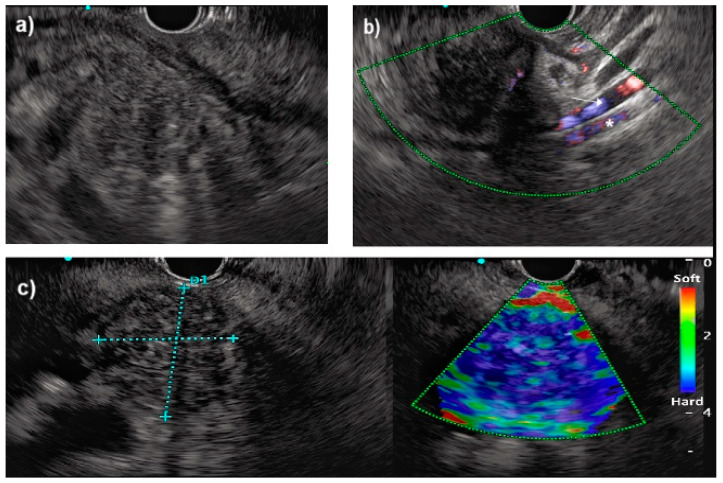
EUS evaluation of pancreatic cancer of the head. (**a**) B-mode evaluation showing hypovascular lesion with irregular margins; (**b**) e-Flow with identification of superior mesenteric artery (star) and venous (arrow); (**c**) EUS elastography showing rigid solid mass suggestive for pancreatic cancer.

Pancreatic ductal adenocarcinoma appears at EUS as an inhomogeneous hypoechoic mass with irregular margins (Figure 3) [10]. Additional techniques allow a better characterization of the lesions such as the application of contrast-enhanced EUS for assessing the vascularization of the mass vascularization or the elastography for estimating the stiffness of the lesion [31,32]. The additional advantages of EUS over cross-sectional images are especially for small pancreatic masses (0.5–2 cm) [10]. For these reasons, EUS is used in association with cross-sectional images in high-risk patients (e.g., subjects with a family history or mutation carriers with/without a family history) as a screening tool to detect early lesions [33]. The main and established role of EUS for the evaluation of PDAC is the ability to perform TA with fine-needle aspiration (FNA) or fine-needle biopsy (FNB). EUS-TA is the current gold standard to obtain histological diagnosis of PDAC compared with percutaneous sampling that is associated with higher adverse events [34]. FNB is generally preferred for TA, but FNA when associated with rapid on-site evaluation (ROSE), has the same sensitivity of FNB for PDAC diagnosis [35]; however, FNA + ROSE is not diffused, it necessitates expert pathologists, and it is more expensive and time consuming [36]. Lastly, EUS-TA also allows an accurate characterization for molecular profiling [37].

Considering the relationship of PDAC with adjacent vessels, EUS has a similar performance compared with CT scans for evaluating tumor vascular invasion and predicting resectability [38]. There are different EUS criteria to predict tumor vascular invasion: the loss of the vessel–parenchymal hyperechoic interface, vessel wall irregularity or luminal narrowing, tumor within the vessel lumen, and the presence of peri-pancreatic venous collaterals in an area of a mass that obliterates the normal anatomic location of a vessel. Different studies showed that EUS may have an higher diagnostic yield than a CT scan to evaluate venous invasion, whereas a CT scan has a better sensitivity to evaluate arterial invasion (of the celiac trunk, superior mesenteric artery, and common hepatic artery) [39,40,41].

Regarding nodal staging, Nawaz et al. published a meta-analysis in which the estimated pooled sensitivity and specificity for nodal invasion were 69% and 81%, respectively [39]. Generally, all the available imaging modalities underestimate nodal metastasis. However, the identification of regional lymph nodes suspicious of metastasis has a significant clinical impact, as these patients will benefit from neoadjuvant therapy [42].

CT scan and MRI remain the best choice for the detection of distant metastasis; however, EUS has a better detection rate of small left hepatic metastatic lesions and small pockets of ascites which could modify management and prognosis in those patients [43].

One retrospective study (n = 42) revealed that 41% of patients were found to have liver metastasis by EUS-FNA that were not previously found on other imaging modalities [19].

## 4. Management According to Resectability Status

### 4.1. Resectable Disease

#### 4.1.1. Endoscopic Biliary Drainage

The role of biliary drainage (BD) is to prevent complications resulting from cholestasis. In patients that are candidates for surgical resection, the major indication for preoperative BD is cholangitis, secondary to biliary obstruction. Other indications are a delayed surgery due to comorbidities requiring preoperative work-up, severe pruritus, and spontaneous coagulopathy [44].

Preoperative BD is also indicated in patients requiring neoadjuvant therapy (NAT). The use of NAT has also been increasing in those patients with a resectable PDAC in order to increase the potential for negative margins and micrometastasis. Furthermore, some chemotherapeutic agents require normalization of liver function [45]. Preoperative BD in patients with asymptomatic jaundice seems not to improve surgical outcomes when surgery is performed in 1–2 weeks [46,47].

Currently, both ERCP and EUS-BD are an effective solution to obtain preoperative BD [48]. ERCP is the historical way to obtain endoscopic BD. The transpapillary drainage allows an internal drainage of the bile and does not alter the anatomy before surgery [44]. Considering the type of stent to use, a lower rate of reintervention and recurrent biliary obstruction was observed with fully-covered metal stents (FC-SEMS), as compared with plastic stents [49,50], with no difference in postoperative outcomes in patients undergoing NAT [51,52].

However, ERCP remains burdened by some complications, such as acute pancreatitis as mentioned before, which has an incidence of 3.5–9.7% and an associated mortality of 0.1–0.7% [53]. Malignant infiltration of duodenum or papilla, as well as modified anatomy that precludes transpapillary stenting, could lead to an augmented incidence of those complications [54]. This type of complication could delay surgery or systemic therapy start, possibly affecting patients’ prognosis.

In the last years, different studies have demonstrated the non-inferiority of EUS-BD, as a first line treatment or after an ERCP failure [55], even if its role in resectable disease remain vague [56].

EUS-BD can be achieved through an extrahepatic access such as choledochoduodenostomy (EUS-CDS) and EUS-gallbladder drainage (EUS-GB) or an intrahepatic approach, with hepaticogastrostomy (EUS-HGS). EUS-CDS is now extensively considered an effective technique. In the setting of pre-operative drainage, EUS-CDS demonstrated high clinical success without impairing surgical outcomes [57]. The first available data came from retrospective series suggesting a technical feasibility and good surgical outcomes [8,58,59], which in certain cases were even better than ERCP [58]. More recently in the “ELEMENT trial”, ten of the 144 included patients underwent pancreatoduodenectomy after biliary drainage (8.2% with EUS-CDS vs. 5.6% with ERCP) and no differences in surgical outcomes were noted [56]. To date, very few data are available about the preoperative role of HGS and EUS-GBD. In a small series of three patients, Koutlas et-al demonstrated the feasibility of pancreatoduodenectomy after HGS [60]. Another Japanese retrospective series on 318 patients undergoing preoperative BD showed the feasibility of HGS with a dedicated plastic stent before surgery on 13 patients [61]. Two case reports also showed the feasibility of Whipple procedure after EUS-GBD [62,63]. In conclusion, in the case of operable patients who need preoperative BD, it is currently recommended to perform ERCP, but when there is a risk of an increased rate of complications for a difficult biliary cannulation, it is possible to perform EUS-CDS without impairing surgical outcomes.

#### 4.1.2. Systemic Therapy (Perioperative Treatment)

Surgery is the selected treatment in resectable PDAC. The survival rate with resection without any systemic adjuvant therapy is very low due to high recurrence rates. Peri-operative and post-operative treatments have the purpose of improving resection rates and survival outcomes (Table 3) [6].

The high postoperative complications rate may delay, reduce, or interrupt the initiation of adjuvant therapy (the actual standard of care in clinical practice with different therapy schemes). The administration of chemotherapy before surgery allows for higher dose-intensity treatment and may improve resection rates. Moreover, in patients with suspicious involvement of locoregional lymph nodes, with large primary tumor or excessive weight loss, NAT is usually suggested in order to improve outcomes [9].

In Japan, the preoperative association of Gemcitabine plus S-1 therapy represents the standard of care for resectable PDAC, according to the results obtained in the PREP2/JSAP5 study, the first trial conducted in neoadjuvant setting [64].

In the SWOGS1505 trial, no significant differences were found between mFOLFIRINOX or Gemcitabine-NabPalitaxel perioperative schedules and neither treatment outperformed the standard of care (i.e., adjuvant treatment), although a greater proportion of patients completed pre-operative treatment compared with patients receiving adjuvant chemotherapy after [65].

The use of neoadjuvant chemoradiation with Gemcitabine followed by Gemcitabine monotherpy in the adjuvant setting produced no clinical benefits in OS compared with standard of care (the primary endpoint of OS was not reached in the PREOPANC-1 trial conducted in the Netherlands), although a minimum benefit in long term OS was observed in particular in patients with borderline resectable disease [66,67].

The most recent international phase II trial was the NORPACT-1 trial which has the purpose of evaluating differences between standard of care (i.e., upfront surgery followed by adjuvant chemotherapy with mFOLFIRINOX for 12 cycles) and perioperative treatment with mFOLFIRINOX (four cycles before and eight cycles after surgery). Partial results do not demonstrate a benefit in 18-month OS (73% in the standard of care group vs. 60% in the experimental group). Despite this, the R0 resection rate was higher in patients treated with perioperative [68].

Adjuvant therapy represents the historical standard of care for patients with resectable and borderline resectable PDAC who underwent to upfront radical surgery. Results of the available trials are discussed in Section 4.2.3.

Several other RCT are ongoing comparing the efficacy of perioperative chemotherapy vs. upfront surgery (PANACE, CAPT-02) or comparing the standard adjuvant therapy with mFOLFIRINOX vs. perioperative mFOLFIRINOX (Alliance A021806, PREOPANC-3).

### 4.2. Borderline Resectable Disease

#### 4.2.1. Endoscopic Biliary Drainage

Borderline resectable tumors are a subgroup of PDAC difficulty in reaching a radical resection (R0). NAT is usually indicated in patients with borderline resectable PDAC. Some chemotherapeutic agents require the normalization of liver function and therefore BD is mandatory in order to avoid biliary complications during NAT (e.g., cholangitis) and to speed up the therapy’s starting.

Borderline resectable PDACs are probably the most difficult category of PDAC to diagnose. The diagnostic process requires adequate time and usually involves the multidisciplinary team. When the patient undergoes EUS-TA for the histological diagnosis, it is usually also offered in the same session an endoscopic BD to relieve patients from jaundice and waiting for the definitive categorization of the PDAC.

There is a paucity of evidence on the best technique to relieve jaundice and/or cholangitis in this specific setting. During ERCP, the choice between plastic stent [69] and SEMS is favorable towards the latter. Three RCT [52,70,71] showed reduced rates of stent occlusion and stent disfunction in the SEMS group without increasing medical costs [71]; one RCT also showed reduced global costs in the SEMS group considering the repeated ERCP in the plastic group [52].

In the “Element Trial”, Chen and colleagues included 38% of borderline resectable/locally advanced patients’ randomized in the ERCP group vs. EUS-CDS and there were no differences in the rate of pancreaticoduodenectomy, operative, and hospitalization time between the two [56].

Drainage technique can influence several factors in borderline resectable tumors, such as time to surgery or to NAT. A multicentre French study retrospectively compared preoperative ERCP or LAMS drainage; interestingly, they found that the times to surgery were shorter in the LAMS group (43 vs. 21 days, *p* = 0.03), and also time to NAT, even if it was not statistically significant (27 vs. 13.5 days, *p* > 0.05) [58].

#### 4.2.2. Systemic Therapy

The current standard of care in borderline resectable PDAC involves the use of NAT with mFOLFIRINOX, in order to obtain a size reduction and a concomitant disease control (Table 4) [6].

The ESPAC 05 study, an RCT, highlighted better results in R0 resection rates with the use of NAT, regardless of the therapy scheme, despite resection rates being higher in the upfront surgery [72]. The mFOLFIRINOX schedule resulted in better R0 resection rates compared with nab-Paclitaxel in association with Gemcitabine, despite no significant differences being observed in [73].

The addition of post-operative chemotherapy with a FOLFOX regimen resulted in better 18-month OS and resection rates, compared with the NAT alone, in the Alliance A021501 phase II trial [74].

Adding radiotherapy in the pre-operative setting has a negative effect [72,74].

In the setting of borderline PDAC, different RCTs are ongoing comparing several chemotherapy regimens. The PANDAS-PRODIGE 44 is a phase II randomized trial conducted in 90 patients with borderline resectable PDSC randomly assigned to receive neoadjuvant FOLFIRINOX with or without concurrent radiotherapy [75]. PREOPANC-2 trial is evaluating neoadjuvant mFOLFIRINOX compared with gemcitabine-based CRT, with both arms receiving gemcitabine adjuvant therapy [76]. The CSPAC-28 trial has the aim of comparing the association of Gemcitabine and nab-Paclitaxel with mFOLFIRINOX in the adjuvant setting in patients with borderline resectable PDAC [77].

#### 4.2.3. Adjuvant Treatment

Adjuvant therapy represents the historical standard of care for patients with resectable and borderline resectable pancreatic disease who underwent upfront radical surgery.

Gemcitabine is the first agent-alone chemotherapy studied in the adjuvant setting for resected PDAC, with confirmed improvement in RFS and OS compared with resection alone in CONKO-001 [78].

In Japan, even S-1 represents one of the standardized post-operative regimens, according to the extremely favorable outcomes observed in the non-inferiority JASPAC-01 trial, compared with Gemcitabine monotherapy [79].

The subsequent introduction of polychemotherapeutic regimens in clinical practice paved the way for new clinical trials not only in metastatic disease but even in this setting.

The ESPAC04 trial represented a turning point in the history of adjuvant treatment in resectable and borderline resectable PDAC. The study compared Gemcitabine with or without Capecitabine as postoperative treatment after resection and showed a statistically significant improvement in overall survival (28.0 vs. 25.5 months, HR 0.82) [80].

In addition, even the results of the PRODIGE-24 clinical trial were revolutionary, demonstrating a relevant benefit in median OS in the adjuvant mFOLFIRINOX group (54.4 months) compared with single-agent Gemcitabine-group (35.0 months). The study even enrolled patients with borderline resectable PDAC [81,82].

Otherwise, the association of Gemcitabine with nab-Paclitaxel compared with Gemcitabine alone did not demonstrate a significant improvement in progression free survival [83].

According to the numerous therapeutic options, the choice of chemotherapy regimen must be guided by general condition, performance status, and comorbidities.

### 4.3. Unresectable Disease

#### 4.3.1. Endoscopic Biliary Drainage

The majority of PDAC patients are not operable at diagnosis. In this setting, almost 80% of patients have jaundice and need a BD. As mentioned before, there are several methods to drain jaundice and ERCP has been considered, in recent years, the standard first line approach. It has been established that the placement of SEMS in this setting allows better outcomes and fewer reinterventions compared with plastic [84]. A recent meta-analysis including 62 studies compared fully covered (FC) SEMS with partial covered (PC) SEMS for the drainage of DMBO, showing a similar rate of AEs but longer times to recurrent biliary obstruction for PC-SEMS [85].

However, obtaining BD during ERCP in DMBO is challenging, especially for the difficulty in obtaining biliary cannulation [86]. Difficult biliary cannulation in this field is also associated with higher rates of AEs. These limitations emphasize the need for alternative ways of obtaining endoscopic BD aside from ERCP.

Several head-to-head studies comparing ERCP and EUS-CDS in this setting showed similar clinical success, rate of AEs, and stent dysfunction [56,87]. Two multicenter RCTs compared EUS-CDS and ERCP as a first line for MDBO showing no differences in stent patency at 1 year or clinical success. Additionally, the procedural time was significantly lower in the EUS group [56,87]. A recent systematic review and meta-analysis has evidenced in EUS-CDS a 96% for both pooled technical and clinical success [88].

Regarding a comparison with the transgastric approach, EUS-CDS is suggested over EUS-HGS, considering that the latter is more technically demanding and is burdened with a higher risk of severe AEs, in particular bile leakage, stent obstruction, and hepatic abscesses [8,48]; however, in situations such as duodenal infiltration and surgical altered anatomy, EUS-HGS allows a rescue solution [89]. Moreover, in the presence of double obstruction (DMBO and gastric outlet obstruction), the association of EUS-HGS and EUS-guided gastroenterostomy have shown better outcomes compared with drainage with EUS-CDS or ERCP for biliary obstruction and with enteral stenting or surgical gastroentero-anastomosis for gastric obstruction [90].

#### 4.3.2. Systemic Therapy

Unresectable disease is related to a metastatic disease or to a locally advanced disease that hamper upfront surgery. In recent years the improvement of diagnostic techniques and surgical skills have made it possible to select patients with apparently unresectable PDAC for induction multi-agent chemotherapy regimens or for concomitant chemoradiation. The NCCN guidelines recommend for patients with locally advanced PDAC without radiological or clinical evidence of distant metastases the use of systemic chemotherapy for 4 or 6 months with induction intent, eventually followed by CRT or stereotactic body radiotherapy in order to make the surgery feasible for patients with no disease progression [9]. It has to be considered that only a small percentage of patients (around 5%) progress to surgery.

The mFOLFIRINOX regimen has proven to be the most appropriate chemotherapy for patients with good performance status and no comorbidities; alongside gemcitabine + nab-paclitaxel, it represents the current standard of care in this setting. The role and the use of radiotherapy under induction in this group of patients is still unclear and needs further studies [91]. Chemoradiation concomitant therapy is considered an option for patients unable to tolerate an induction regimen with multiple agent chemotherapy.

Gemcitabine plus nab-paclitaxel showed promising effects on overall survival (OS) and progression-free survival (PFS) when compared with the gemcitabine monotherapy in the MPACT trial [92]. Similarly, in the PRODIGE4/ACCORD11 trial, the FOLFIRINOX regimen improved survival rates compared with gemcitabine monotherapy [93], which could be considered an option only for patients with comorbidities that contraindicate combination treatments. No statistical difference was found in increasing the OS between the two regimens in several meta-analysis [94,95,96].

In the NAPOLI-3 randomized phase 3 trial, the NALIRIFOX regimen (combination of oxaliplatin, liposomal irinotecan, fluorouracil, and leucovorin) was compared with the nab-paclitaxel and gemcitabine combination as first-line chemotherapy for PDAC, highlighting a statistically significant improvement in OS and PFS [97].

The most recent research has focused on the molecular profile of PC, highlighting biomarkers and mutations possibly associated with the development and progression of PC, such as KRAS, MSI, TMB-H, BRAF, NTRK, and RET [98,99]. This contributed to better identifying potential new drugs for metastatic disease.

More than 90% of patients with metastatic pancreatic cancer harbor KRAS somatic mutations, in particular single point mutations at codon 12 are the most common. Until now, selective inhibitors have been developed only for KRAS G12C mutations and some early phase basket trials have shown encouraging results also for metastatic PC [100,101].

Further studies are needed to understand new potential therapeutic targets as well as the role of new agents in the treatment landscape of metastatic pancreatic cancer.

### 4.4. Controversies on Resectability Status

**Figure 4:**
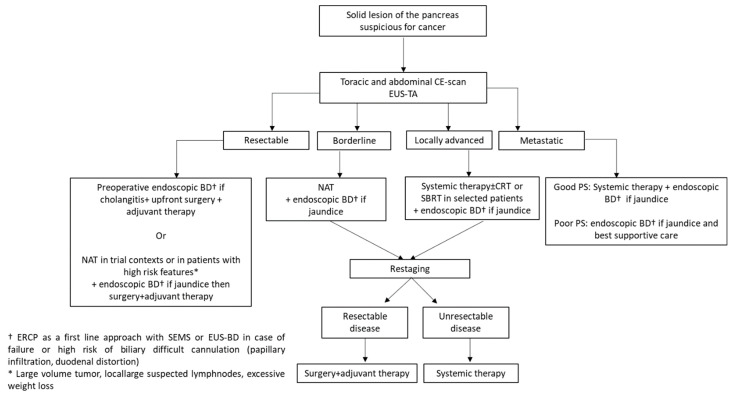
Practical algorithm for the management of pancreatic cancer. CE, contrast-enhanced; EUS-TA, endoscopic ultrasound, tissue acquisition; BD, biliary drainage; NAT, neoadjuvant therapy; CRT, Chemo-radio therapy; SBRT, stereotactic body radiotherapy.

As mentioned before, the staging and resectability criteria for PDAC are uniform and standardized in most countries and regions of the world; however, there are some ongoing controversies about the resectability status (Figure 4).

One such issue is the histopathological assessment of the R-status after surgery, defined as a distance of the tumor from the resection margin of ≤1 mm. Several histopathological evaluations have been used in the past and it was shown that the use of the Leeds protocol was related to higher R1-status [102,103]. The Leeds protocol is multicolour margin staining with axial slicing and extensive tissue sampling; specifically, the axial slicing method obtains perpendicular sections for all the relevant soft tissue margins and can easily evaluate the distance between resection margin and tumor. In a high-volume center performing pancreaticoduodenectomy, the use of the Leeds protocol increased the R1-status rate from 14% to 76% [102].

The involvement of CA > 180° is generally considered not suitable for first-line surgery and it is also generally associated with the involvement of aorta and SM. However, there is a debate about whether all patients with CA involvement should be categorized as unresectable. A small subgroup of patients with CA involvement, without involvement of the SMA or aorta, may benefit from distal pancreatectomy with celiac axis resection (DP-CAR), which has been shown to be both safe and effective. Studies suggest that DP-CAR does not result in higher complications, mortality, or reduced overall survival compared with distal pancreatectomy in cases where the CA is not involved [104].

## 5. Conclusions

The management of patients with PDAC is continuously evolving with a progressive improvement of survival in the recent years. The availability of several chemotherapeutic protocols and ongoing trials allows a wide choice of treatment that should be personalized according to the staging of PDAC and patients’ performance status. Moreover, the increased availability and efficacy of different endoscopic BD procedures needs an accurate selection of the technique in a multidisciplinary setting in order to avoid perioperative AEs and to allow a faster referral for systemic therapies.

## Figures and Tables

**Table 1:** TNM staging system of pancreatic cancer by American Joint Commettee on Cancer (AJCC), 8th edition (2017).

Primary Tumor (T)		
TX	Primary tumor cannot be assessed	
T0	No evidence of primary tumor	
Tis	Carcinoma in situ	
T1	Tumors ≤ 2 cm in greatest dimension	
	T1a	Tumors ≤ 0.5 cm in greatest dimension
	T1b	Tumor > 0.5 cm and <1 cm in greatest dimension
	T1c	Tumor 1–2 cm in greatest dimension
T2	Tumor > 2 cm and ≤4 cm in greatest dimension	
T3	Tumor > 4 cm in greatest dimension	
T4	Tumor involves the celiac axis, superior mesenteric artery, and/or common hepatic artery, regardless of size	
**Regional Lymph Nodes (N)**		
NX	Regional lymph node cannot be assessed	
N0	No regional lymph node metastases	
N1	Metastasis in 1–3 regional lymph nodes	
N2	Metastasis in ≥4 regional lymph nodes	
**Distant metastasis**		
M0	No distant metastasis	
M1	Distant metastasis	

**Table 2:** Resectability anatomical criteria by National Comprehensive Cancer Network (NCCN), April 2024.

Resectability Status	Arterial	Venous
**Resectable**	- No arterial tumor contact (CA, SMA, or CHA).	- No tumor contacts with the SMV or PV or ≤180° contact without vein contour irregularity.
**Borderline Resectable**	**Pancreatic head/uncinate process:** - Solid tumor contact with CHA without extension to CA or hepatic artery bifurcation allowing for safe and complete resection and reconstruction. - Solid tumor contact with the SMA of ≤180°. - Solid tumor contact with variant arterial anatomy (e.g., accessory right hepatic artery, replaced right hepatic artery, replaced CHA, and the origin of replaced or accessory artery) and the presence and degree of tumor contact should be noted if present, as it may affect surgical planning. **Pancreatic body/tail:** Solid tumor contact with the CA of ≤180°.	- Solid tumor contact with the SMV or PV of >180°, contact of ≤180° with contour irregularity of the vein or thrombosis of the vein but with suitable vessel proximal and distal to the site of involvement allowing for safe and complete resection and vein reconstruction. - Solid tumor contact with the inferior vena cava (IVC).
**Locally Advanced**	**Head/uncinate process:** Solid tumor contact > 180° with the SMA or CA. **Pancreatic body/tail:** - Solid tumor contact of >180° with the SMA or CA. - Solid tumor contact with the CA and aortic involvement.	- Unreconstructible SMV/PV due to tumor involvement or occlusion (can be due to tumor or bland thrombus)

CA, celiac axis; SMA, superior mesenteric artery; CHA, common hepatic artery; SMV, superior mesenteric vein; PV, portal vein.

**Table 3:** Current available studies of perioperative treatment in resectable pancreatic cancer.

Study	Phase	Year	Intervention	Resection Rate	R0 Resection Rate	Primary Outcome
PREP2/JSAP5	II/III	2019	Gemcitabine + S-1	-	-	OS: 36.7 vs. 26.6 mo
SWOGS1505	II	2021	mFOLFIRINOX vs. Gemcitabine + nab-Paclitaxel	73% vs. 70%	61.8% vs. 59.6%	OS: 23.2 vs. 23.6 mo
PREOPANC-1	III	2023	Gemcitabine + concomitant RT (36 Gy)	69.5% vs. 72.4% (upfront surgery)	41.2% vs. 26.8% (upfront surgery)	OS: 15.7 vs. 14.3 mo
NORPACT-1	II	2024	mFOLFIRINOX	82% vs. 89% (upfront surgery)	56% vs. 39% (upfront surgery)	18-mo OS: 60% vs. 73% (upfront surgery)

OS, overall survival, mFOLFIRINOX (irinotecan 150 mg/m^2^ day^−1^, oxaliplatin 85 mg/m^2^ day^−1^, leucovorin 400 mg/m^2^ day^−1^, 5-fluorouracil (5-FU) 2400 mg/m^2^ over 46 h every 2 weeks for 12 cycles).

**Table 4:** Current available studies of perioperative treatment in borderline resectable pancreatic cancer.

Study	Phase	Year	Intervention	Resection Rate	R0 Resection Rate	Primary Outcome
Alliance A021501	II	2022	mFOLFIRINOX +/−RT (+adiuvant FOLFOX)	49% vs. 35%	-	18-mo OS: 66.7% vs. 47.3%
ESPAC 05	II	2023	Gemcitabina plus Capecitabina or FOLIRINOX or Capecitabine plus RT	55% (intervention groups combined) vs. 68% (upfront surgery)	23% (intervention groups combined) vs. 14% (upfront surgery)	Resection rate
NUPAT 01	II	2022	FOLFIRINOX vs. Gemcitabine plus nab-Paclitaxel	88.5% vs. 88%	73.1% vs. 56%	R0 resection rate

## References

[1] Klein A.P. (2021). Pancreatic cancer epidemiology: Understanding the role of lifestyle and inherited risk factors. Nat. Rev. Gastroenterol. Hepatol..

[2] Huang J., Lok V., Ngai C.H., Zhang L., Yuan J., Lao X.Q., Ng K., Chong C., Zheng Z.-J., Wong M.C. (2021). Worldwide Burden of, Risk Factors for, and Trends in Pancreatic Cancer. Gastroenterology.

[3] Chatterjee A., Shah J. (2023). Role of Endoscopic Ultrasound in Diagnosis of Pancreatic Ductal Adenocarcinoma. Diagnostics.

[4] Soloff E.V., Zaheer A., Meier J., Zins M., Tamm E.P. (2018). Staging of pancreatic cancer: Resectable, borderline resectable, and unresectable disease. Abdom. Radiol..

[5] Stoffel E.M., Brand R.E., Goggins M. (2023). Pancreatic Cancer: Changing Epidemiology and New Approaches to Risk Assessment, Early Detection, and Prevention. Gastroenterology.

[6] Conroy T., Pfeiffer P., Vilgrain V., Lamarca A., Seufferlein T., O’reilly E., Hackert T., Golan T., Prager G., Haustermans K. (2023). Pancreatic cancer: ESMO Clinical Practice Guideline for diagnosis, treatment and follow-up. Ann. Oncol..

[7] Dumonceau J.M., Tringali A., Papanikolaou I.S., Blero D., Mangiavillano B., Schmidt A., van Hooft J.E. (2018). Endoscopic biliary stenting: Indications, choice of stents, and results: European Society of Gastrointestinal Endoscopy (ESGE) Clinical Guideline-Updated October 2017. Endoscopy.

[8] Fabbri C., Scalvini D., Paolo G., Binda C., Mauro A., Coluccio C., Mazza S., Trebbi M., Viera F.T., Anderloni A. (2024). Complications and management of interventional endoscopic ultrasound: A critical review. Best Pract. Res. Clin. Gastroenterol..

[9] Tempero M.A., Malafa M.P., Al-Hawary M., Behrman S.W., Benson A.B., Cardin D.B., George G.V. (2021). Pancreatic Adenocarcinoma, Version 2.2021, NCCN Clinical Practice Guidelines in Oncology. J. Natl. Compr. Cancer Netw..

[10] Yousaf M.N., Chaudhary F.S., Ehsan A., Suarez A.L., Muniraj T., Jamidar P., Aslanian H.R., Farrell J.J. (2020). Endoscopic ultrasound (EUS) and the management of pancreatic cancer. BMJ Open Gastroenterol..

[11] Balachandran A., Bhosale P.R., Charnsangavej C., Tamm E.P. (2014). Imaging of pancreatic neoplasms. Surg. Oncol. Clin. North Am..

[12] Alexakis N., Halloran C., Raraty M., Ghaneh P., Sutton R., Neoptolemos J.P. (2004). Current standards of surgery for pancreatic cancer. Br. J. Surg..

[13] Varadhachary G.R., Tamm E.P., Abbruzzese J.L., Xiong H.Q., Crane C.H., Wang H., Lee J.E., Pisters P.W.T., Evans D.B., Wolff R.A. (2006). Borderline resectable pancreatic cancer: Definitions, management, and role of preoperative therapy. Ann. Surg. Oncol..

[14] Lu R., Luthra A., Han S. (2022). Combined EUS-guided gallbladder drainage with rendezvous ERCP for treatment of concomitant cholecystitis, cholelithiasis, and choledocholithiasis. VideoGIE.

[15] Hough T.J., Raptopoulos V., Siewert B., Matthews J.B. (1999). Teardrop superior mesenteric vein: CT sign for unresectable carcinoma of the pancreas. AJR Am. J. Roentgenol..

[16] Javed A.A., Ding D., Baig E., Wright M.J., Teinor J.A., Mansoor D., Thompson E., Hruban R.H., Narang A., Burns W.R. (2022). Accurate Nodal Staging in Pancreatic Cancer in the Era of Neoadjuvant Therapy. World J. Surg..

[17] Koelblinger C., Ba-Ssalamah A., Goetzinger P., Puchner S., Weber M., Sahora K., Schima W. (2011). Gadobenate dimeglumine-enhanced 3.0-T MR imaging versus multiphasic 64-detector row CT: Prospective evaluation in patients suspected of having pancreatic cancer. Radiology.

[18] Motosugi U., Ichikawa T., Morisaka H., Sou H., Muhi A., Kimura K., Sano K., Araki T. (2011). Detection of pancreatic carcinoma and liver metastases with gadoxetic acid-enhanced MR imaging: Comparison with contrast-enhanced multi-detector row CT. Radiology.

[19] Okasha H., Wifi M.-N., Awad A., Abdelfatah Y., Abdelfatah D., El-Sawy S., Alzamzamy A., Abou-Elenin S., Abou-Elmagd A., ElHusseiny R. (2021). Role of EUS in detection of liver metastasis not seen by computed tomography or magnetic resonance imaging during staging of pancreatic, gastrointestinal, and thoracic malignancies. Endosc. Ultrasound.

[20] Wong J.C., Lu D.S. (2008). Staging of pancreatic adenocarcinoma by imaging studies. Clin. Gastroenterol. Hepatol..

[21] A Bluemke D., Cameron J.L., Hruban R.H., A Pitt H., Siegelman S.S., Soyer P., Fishman E.K. (1995). Potentially resectable pancreatic adenocarcinoma: Spiral CT assessment with surgical and pathologic correlation. Radiology.

[22] Fusari M., Maurea S., Imbriaco M., Mollica C., Avitabile G., Soscia F., Camera L., Salvatore M. (2010). Comparison between multislice CT and MR imaging in the diagnostic evaluation of patients with pancreatic masses. Radiol. Med..

[23] Legmann P., Vignaux O., Dousset B., Baraza A.J., Palazzo L., Dumontier I., Coste J., Louvel A., Roseau G., Couturier D. (1998). Pancreatic tumors: Comparison of dual-phase helical CT and endoscopic sonography. AJR Am. J. Roentgenol..

[24] DeWitt J., Devereaux B., Chriswell M., McGreevy K., Howard T., Imperiale T.F., Ciaccia D., Lane K.A., Maglinte D., Kopecky K. (2004). Comparison of endoscopic ultrasonography and multidetector computed tomography for detecting and staging pancreatic cancer. Ann. Intern. Med..

[25] Baron R.L. (1994). Understanding and optimizing use of contrast material for CT of the liver. AJR Am. J. Roentgenol..

[26] Sahani D.V., Shah Z.K., A Catalano O., Boland G.W., Brugge W.R. (2008). Radiology of pancreatic adenocarcinoma: Current status of imaging. J. Gastroenterol. Hepatol..

[27] Fletcher J.G., Wiersema M.J., Farrell M.A., Fidler J.L., Burgart L.J., Koyama T., Harmsen W.S. (2003). Pancreatic malignancy: Value of arterial, pancreatic, and hepatic phase imaging with multi-detector row CT. Radiology.

[28] Golse N., Lebeau R., Lombard-Bohas C., Hervieu V., Ponchon T., Adham M. (2013). Lymph node involvement beyond peripancreatic region in pancreatic head cancers: When results belie expectations. Pancreas.

[29] Kim J.H., Park S.H., Yu E.S., Kim M.H., Kim J., Byun J.H., Lee M.G. (2010). Visually isoattenuating pancreatic adenocarcinoma at dynamic-enhanced CT: Frequency, clinical and pathologic characteristics, and diagnosis at imaging examinations. Radiology.

[30] Wang Y., Miller F.H., Chen Z.E., Merrick L., Mortele K.J., Hoff F.L., Hammond N.A., Yaghmai V., Nikolaidis P. (2011). Diffusion-weighted MR imaging of solid and cystic lesions of the pancreas. Radiographics.

[31] Mei M., Ni J., Liu D., Jin P., Sun L. (2013). EUS elastography for diagnosis of solid pancreatic masses: A meta-analysis. Gastrointest Endosc..

[32] Kitano M., Kudo M., Yamao K., Takagi T., Sakamoto H., Komaki T., Kamata K., Imai H., Chiba Y., Okada M. (2012). Characterization of small solid tumors in the pancreas: The value of contrast-enhanced harmonic endoscopic ultrasonography. Am. J. Gastroenterol..

[33] Yang J., Xu R., Wang C., Qiu J., Ren B., You L. (2021). Early screening and diagnosis strategies of pancreatic cancer: A comprehensive review. Cancer Commun..

[34] Horwhat J.D., Paulson E.K., McGrath K., Branch M.S., Baillie J., Tyler D., Pappas T., Enns R., Robuck G., Stiffler H. (2006). A randomized comparison of EUS-guided FNA versus CT or US-guided FNA for the evaluation of pancreatic mass lesions. Gastrointest. Endosc..

[35] Gkolfakis P., Crinò S.F., Tziatzios G., Ramai D., Papaefthymiou A., Papanikolaou I.S., Triantafyllou K., Arvanitakis M., Lisotti A., Fusaroli P. (2022). Comparative diagnostic performance of end-cutting fine-needle biopsy needles for EUS tissue sampling of solid pancreatic masses: A network meta-analysis. Gastrointest. Endosc..

[36] Zakaria A., Al-Share B., Klapman J.B., Dam A. (2022). The Role of Endoscopic Ultrasonography in the Diagnosis and Staging of Pancreatic Cancer. Cancers.

[37] Bang J.Y., Jhala N., Seth A., Krall K., Navaneethan U., Hawes R., Wilcox C.M., Varadarajulu S. (2023). Standardisation of EUS-guided FNB technique for molecular profiling in pancreatic cancer: Results of a randomised trial. Gut.

[38] James P.D., Meng Z.W., Zhang M., Belletrutti P.J., Mohamed R., Ghali W., Roberts D.J., Martel G., Heitman S.J. (2017). The incremental benefit of EUS for identifying unresectable disease among adults with pancreatic adenocarcinoma: A meta-analysis. PLoS ONE.

[39] Nawaz H., Fan C.Y., Kloke J., Khalid A., McGrath K., Landsittel D., I Papachristou G. (2013). Performance characteristics of endoscopic ultrasound in the staging of pancreatic cancer: A meta-analysis. Jop.

[40] Bispo M., Marques S., Rio-Tinto R., Fidalgo P., Devière J. (2021). The Role of Endoscopic Ultrasound in Pancreatic Cancer Staging in the Era of Neoadjuvant Therapy and Personalised Medicine. GE Port J. Gastroenterol..

[41] Li J.-H., He R., Li Y.-M., Cao G., Ma Q.-Y., Yang W.-B. (2014). Endoscopic ultrasonography for tumor node staging and vascular invasion in pancreatic cancer: A meta-analysis. Dig. Surg..

[42] Isaji S., Mizuno S., Windsor J.A., Bassi C., Castillo C.F.-D., Hackert T., Hayasaki A., Katz M.H., Kim S.-W., Kishiwada M. (2018). International consensus on definition and criteria of borderline resectable pancreatic ductal adenocarcinoma 2017. Pancreatology.

[43] Nguyen P.T., Chang K.J. (2001). EUS in the detection of ascites and EUS-guided paracentesis. Gastrointest. Endosc..

[44] Nehme F., Lee J.H. (2022). Preoperative biliary drainage for pancreatic cancer. Dig. Endosc..

[45] Varadhachary G.R., Wolff R.A., Crane C.H., Sun C.C., Pisters P.W., Vauthey J.-N., Abdalla E., Wang H., Staerkel G.A., Lee J.H. (2008). Preoperative gemcitabine and cisplatin followed by gemcitabine-based chemoradiation for resectable adenocarcinoma of the pancreatic head. J. Clin. Oncol..

[46] van der Gaag N.A., Rauws E.A., van Eijck C.H., Bruno M.J., van der Harst E., Kubben F.J., Gouma D.J. (2010). Preoperative biliary drainage for cancer of the head of the pancreas. N. Engl. J. Med..

[47] Shen Z., Zhang J., Chen H., Wang W., Xu W., Lu X., Shen B. (2020). Does Pre-operative Biliary Drainage Influence Long-Term Survival in Patients With Obstructive Jaundice With Resectable Pancreatic Head Cancer?. Front. Oncol..

[48] Marzioni M., Crinò S.F., Lisotti A., Fuccio L., Vanella G., Amato A., Bertani H., Binda C., Coluccio C., Forti E. (2024). Biliary drainage in patients with malignant distal biliary obstruction: Results of an Italian consensus conference. Surg. Endosc..

[49] Song T.J., Lee J.H., Lee S.S., Jang J.W., Kim J.W., Ok T.J., Oh D.W., Park D.H., Seo D.W., Lee S.K. (2016). Metal versus plastic stents for drainage of malignant biliary obstruction before primary surgical resection. Gastrointest. Endosc..

[50] Mandai K., Tsuchiya T., Kawakami H., Ryozawa S., Saitou M., Iwai T., Ogawa T., Tamura T., Doi S., Okabe Y. (2022). Fully covered metal stents vs. plastic stents for preoperative biliary drainage in patients with resectable pancreatic cancer without neoadjuvant chemotherapy: A multicenter, prospective, randomized controlled trial. J. Hepato-Biliary-Pancreat. Sci..

[51] Lyu Y., Ye S., Wang B. (2023). Comparison of metal versus plastic stent for preoperative biliary drainage in patients with pancreatic cancer undergoing neoadjuvant therapy: A meta-analysis and systematic review. BMC Gastroenterol..

[52] Gardner T.B., Spangler C.C., Byanova K.L., Ripple G.H., Rockacy M.J., Levenick J.M., Smith K.D., Colacchio T.A., Barth R.J., Zaki B.I. (2016). Cost-effectiveness and clinical efficacy of biliary stents in patients undergoing neoadjuvant therapy for pancreatic adenocarcinoma in a randomized controlled trial. Gastrointest Endosc..

[53] Dumonceau J.-M., Kapral C., Aabakken L., Papanikolaou I.S., Tringali A., Vanbiervliet G., Beyna T., Dinis-Ribeiro M., Hritz I., Mariani A. (2020). ERCP-related adverse events: European Society of Gastrointestinal Endoscopy (ESGE) Guideline. Endoscopy.

[54] Wu C.C.H., Lim S.J.M., Khor C.J.L. (2023). Endoscopic retrograde cholangiopancreatography-related complications: Risk stratification, prevention, and management. Clin. Endosc..

[55] Fugazza A., Fabbri C., Di Mitri R., Petrone M.C., Colombo M., Cugia L., Amato A., Forti E., Binda C., Maida M. (2022). EUS-guided choledochoduodenostomy for malignant distal biliary obstruction after failed ERCP: A retrospective nationwide analysis. Gastrointest. Endosc..

[56] Chen Y.-I., Sahai A., Donatelli G., Lam E., Forbes N., Mosko J., Paquin S.C., Donnellan F., Chatterjee A., Telford J. (2023). Endoscopic Ultrasound-Guided Biliary Drainage of First Intent With a Lumen-Apposing Metal Stent vs Endoscopic Retrograde Cholangiopancreatography in Malignant Distal Biliary Obstruction: A Multicenter Randomized Controlled Study (ELEMENT Trial). Gastroenterology.

[57] Fabbri C., Fugazza A., Binda C., Zerbi A., Jovine E., Cennamo V., Repici A., Anderloni A. (2019). Beyond palliation: Using EUS-guided choledochoduodenostomy with a lumen-apposing metal stent as a bridge to surgery. a case series. J. Gastrointestin. Liver Dis..

[58] Janet J., Albouys J., Napoleon B., Jacques J., Mathonnet M., Magne J., Fontaine M., de Ponthaud C., Fontanier S.D., Bardet S.S.M. (2023). Pancreatoduodenectomy Following Preoperative Biliary Drainage Using Endoscopic Ultrasound-Guided Choledochoduodenostomy Versus a Transpapillary Stent: A Multicenter Comparative Cohort Study of the ACHBT-FRENCH-SFED Intergroup. Ann. Surg. Oncol..

[59] Gaujoux S., Jacques J., Bourdariat R., Sulpice L., Lesurtel M., Truant S., Robin F., Prat F., Palazzo M., Schwarz L. (2021). Pancreaticoduodenectomy following endoscopic ultrasound-guided choledochoduodenostomy with electrocautery-enhanced lumen-apposing stents an ACHBT-SFED study. HPB.

[60] Koutlas N.J., LePage E.M., Lucioni T., Pawa S., Pawa R. (2022). Preoperative Endoscopic Ultrasound-Guided Hepaticogastrostomy Facilitates Decompression and Diagnosis in Patients With Suspected Malignant Biliary Obstruction: A Case Series. Cureus.

[61] Mukai S., Itoi T., Tsuchiya T., Ishii K., Tonozuka R., Nagakawa Y., Kozono S., Takishita C., Osakabe H., Sofuni A. (2023). Clinical feasibility of endoscopic ultrasound-guided biliary drainage for preoperative management of malignant biliary obstruction (with videos). J. Hepato-Biliary-Pancreat. Sci..

[62] Ligresti D., Tarantino I., Amata M., Cipolletta F., Gruttadauria S., Cintorino D., Traina M. (2019). Bridge-to-surgery gallbladder drainage with a lumen-apposing metal stent in malignant distal biliary obstruction: A choice tailored for the surgeon. Endoscopy.

[63] Lariño-Noia J., Fernández R.M., Novo M.P., García D.d.l.I., Iglesias-García J., Castiñeira A.Q., Pérez E.V., Dominguez-Muñoz J.E. (2022). Emergent endoscopic ultrasound-guided cholecystoduodenostomy does not prevent R0 resection in a pancreaticoduodenectomy for pancreatic cancer. Clin. J. Gastroenterol..

[64] Motoi F., Kosuge T., Ueno H., Yamaue H., Satoi S., Sho M. (2019). Randomized phase II/III trial of neoadjuvant chemotherapy with gemcitabine and S-1 versus upfront surgery for resectable pancreatic cancer (Prep-02/JSAP05). Jpn. J. Clin. Oncol..

[65] Ahmad S.A., Duong M., Sohal D.P.S., Gandhi N.S., Beg M.S., Wang-Gillam A., Wade J.L., Chiorean E.G., Guthrie K.A., Lowy A.M. (2020). Surgical Outcome Results From SWOG S1505: A Randomized Clinical Trial of mFOLFIRINOX Versus Gemcitabine/Nab-paclitaxel for Perioperative Treatment of Resectable Pancreatic Ductal Adenocarcinoma. Ann. Surg..

[66] Versteijne E., Suker M., Groothuis K., Akkermans-Vogelaar J.M., Besselink M.G., Bonsing B.A. (2020). Preoperative Chemoradiotherapy Versus Immediate Surgery for Resectable and Borderline Resectable Pancreatic Cancer: Results of the Dutch Randomized Phase III PREOPANC Trial. J. Clin. Oncol..

[67] Versteijne E., van Dam J.L., Suker M., Janssen Q.P., Groothuis K., Akkermans-Vogelaar J.M., Besselink M.G., Bonsing B.A., Buijsen J., Busch O.R. (2022). Neoadjuvant Chemoradiotherapy Versus Upfront Surgery for Resectable and Borderline Resectable Pancreatic Cancer: Long-Term Results of the Dutch Randomized PREOPANC Trial. J. Clin. Oncol..

[68] Labori K.J., Bratlie S.O., Andersson B., Angelsen J.-H., Biörserud C., Björnsson B., Bringeland E.A., Elander N., Garresori H., Grønbech J.E. (2024). Neoadjuvant FOLFIRINOX versus upfront surgery for resectable pancreatic head cancer (NORPACT-1): A multicentre, randomised, phase 2 trial. Lancet Gastroenterol. Hepatol..

[69] Van Steenbergen W. (2001). Treatment of malignant biliary stenosis: Which stent to use?. Acta Gastroenterol Belg.

[70] Seo D.W., Sherman S., Dua K.S., Slivka A., Roy A., Costamagna G., Deviere J., Peetermans J., Rousseau M., Nakai Y. (2019). Covered and uncovered biliary metal stents provide similar relief of biliary obstruction during neoadjuvant therapy in pancreatic cancer: A randomized trial. Gastrointest. Endosc..

[71] Tamura T., Itonaga M., Ashida R., Yamashita Y., Hatamaru K., Kawaji Y., Emori T., Kitahata Y., Miyazawa M., Hirono S. (2021). Covered self-expandable metal stents versus plastic stents for preoperative biliary drainage in patient receiving neo-adjuvant chemotherapy for borderline resectable pancreatic cancer: Prospective randomized study. Dig. Endosc..

[72] Ghaneh P., Palmer D., Cicconi S., Jackson R., Halloran C.M., Rawcliffe C., Sripadam R., Mukherjee S., Soonawalla Z., Wadsley J. (2023). Immediate surgery compared with short-course neoadjuvant gemcitabine plus capecitabine, FOLFIRINOX, or chemoradiotherapy in patients with borderline resectable pancreatic cancer (ESPAC5): A four-arm, multicentre, randomised, phase 2 trial. Lancet Gastroenterol. Hepatol..

[73] Katz M.H., Shi Q., Meyers J., Herman J.M., Chuong M., Wolpin B.M., O’Reilly E.M. (2022). Efficacy of Preoperative mFOLFIRINOX vs mFOLFIRINOX Plus Hypofractionated Radiotherapy for Borderline Resectable Adenocarcinoma of the Pancreas: The A021501 Phase 2 Randomized Clinical Trial. JAMA Oncol..

[74] Katz M.H., Shi Q., Meyers J., Herman J.M., Chuong M., Wolpin B.M., O’Reilly E.M. (2022). Results of a Phase II Study on the Use of Neoadjuvant Chemotherapy (FOLFIRINOX or GEM/nab-PTX) for Borderline-resectable Pancreatic Cancer (NUPAT-01). Ann. Surg..

[75] Schwarz L., Vernerey D., Bachet J.-B., Tuech J.-J., Portales F., Michel P., Cunha A.S. (2018). Resectable pancreatic adenocarcinoma neo-adjuvant FOLF(IRIN)OX-based chemotherapy-a multicenter, non-comparative, randomized, phase II trial (PANACHE01-PRODIGE48 study). BMC Cancer.

[76] Janssen Q.P., van Dam J.L., Bonsing B.A., Bos H., Bosscha K.P., Coene P.P.L.O., van Eijck C.H.J., de Hingh I.H.J.T., Karsten T.M., van der Kolk M.B. (2021). Total neoadjuvant FOLFIRINOX versus neoadjuvant gemcitabine-based chemoradiotherapy and adjuvant gemcitabine for resectable and borderline resectable pancreatic cancer (PREOPANC-2 trial): Study protocol for a nationwide multicenter randomized controlled trial. BMC Cancer.

[77] Yu X.-J. https://clinicaltrials.gov/study/NCT04617821.

[78] Oettle H., Neuhaus P., Hochhaus A., Hartmann J.T., Gellert K., Ridwelski K., Riess H. (2013). Adjuvant chemotherapy with gemcitabine and long-term outcomes among patients with resected pancreatic cancer: The CONKO-001 randomized trial. JAMA.

[79] Uesaka K., Boku N., Fukutomi A., Okamura Y., Konishi M., Matsumoto I., Kaneoka Y., Shimizu Y., Nakamori S., Sakamoto H. (2016). Adjuvant chemotherapy of S-1 versus gemcitabine for resected pancreatic cancer: A phase 3, open-label, randomised, non-inferiority trial (JASPAC 01). Lancet.

[80] Neoptolemos J.P., Palmer D.H., Ghaneh P., Psarelli E.E., Valle J.W., Halloran C.M., Faluyi O., O’Reilly D.A., Cunningham D., Wadsley J. (2017). Comparison of adjuvant gemcitabine and capecitabine with gemcitabine monotherapy in patients with resected pancreatic cancer (ESPAC-4): A multicentre, open-label, randomised, phase 3 trial. Lancet.

[81] Conroy T., Hammel P., Hebbar M., Ben Abdelghani M., Wei A.C., Raoul J.-L., Choné L., Francois E., Artru P., Biagi J.J. (2018). FOLFIRINOX or Gemcitabine as Adjuvant Therapy for Pancreatic Cancer. N. Engl. J. Med..

[82] Conroy T., Castan F., Lopez A., Turpin A., Abdelghani M.B., Wei A.C., O’Callaghan C. (2022). Five-Year Outcomes of FOLFIRINOX vs Gemcitabine as Adjuvant Therapy for Pancreatic Cancer: A Randomized Clinical Trial. JAMA Oncol..

[83] Tempero M.A., Pelzer U., O’Reilly E.M., Winter J., Oh D.Y., Li C.P., Reni M. (2023). Adjuvant nab-Paclitaxel + Gemcitabine in Resected Pancreatic Ductal Adenocarcinoma: Results From a Randomized, Open-Label, Phase III Trial. J. Clin. Oncol..

[84] Elshimi E., Morad W., Elshaarawy O., Attia A. (2020). Optimization of biliary drainage in inoperable distal malignant strictures. World J. Gastrointest. Endosc..

[85] Vanella G., Coluccio C., Cucchetti A., Leone R., Dell’anna G., Giuffrida P., Abbatiello C., Binda C., Fabbri C., Arcidiacono P.G. (2024). Fully covered versus partially covered self-expandable metal stents for palliation of distal malignant biliary obstruction: A systematic review and meta-analysis. Gastrointest. Endosc..

[86] Anderloni A., Fugazza A., Spadaccini M., Colombo M., Gabbiadini R., Siracusano L., Pressiani T., Repici A. (2021). Single-session EUS-guided gastroenterostomy and hepaticogastrostomy using dedicated metal stents (with videos). Endosc. Ultrasound.

[87] Teoh A.Y.B., Napoleon B., Kunda R., Arcidiacono P.G., Kongkam P., Larghi A., Van der Merwe S., Jacques J., Legros R., Thawee R.-E. (2023). EUS-Guided Choledocho-duodenostomy Using Lumen Apposing Stent Versus ERCP With Covered Metallic Stents in Patients With Unresectable Malignant Distal Biliary Obstruction: A Multicenter Randomized Controlled Trial (DRA-MBO Trial). Gastroenterology.

[88] Fugazza A., Khalaf K., Spadaccini M., Facciorusso A., Colombo M., Andreozzi M., Carrara S., Binda C., Fabbri C., Anderloni A. (2024). Outcomes predictors in endoscopic ultrasound-guided choledochoduodenostomy with lumen-apposing metal stent: Systematic review and meta-analysis. Endosc. Int. Open.

[89] Vanella G., Bronswijk M., van Wanrooij R.L., Dell’Anna G., Laleman W., van Malenstein H., Voermans R.P., Fockens P., Van der Merwe S., Arcidiacono P.G. (2023). Combined endoscopic mAnagement of BiliaRy and gastrIc OutLET obstruction (CABRIOLET Study): A multicenter retrospective analysis. DEN Open.

[90] Vanella G., Leone R., Frigo F., Bronswijk M., van Wanrooij R.L.J., Tamburrino D., Orsi G., Belfiori G., Macchini M., Reni M. (2025). Endoscopic ultrasound-guided choledochoduodenostomy versus hepaticogastrostomy combined with gastroenterostomy in malignant double obstruction (CABRIOLET_Pro): A prospective comparative study. DEN Open.

[91] Fietkau R., Grützmann R., Wittel U.A., Croner R.S., Jacobasch L., Neumann U.P., Reinacher-Schick A., Imhoff D., Boeck S., Keilholz L. (2021). R0 resection following chemo (radio)therapy improves survival of primary inoperable pancreatic cancer patients. Interim results of the German randomized CONKO-007± trial. Strahlenther. Onkol..

[92] Von Hoff D.D., Ervin T., Arena F.P., Chiorean E.G., Infante J., Moore M., Renschler M.F. (2013). Increased survival in pancreatic cancer with nab-paclitaxel plus gemcitabine. N. Engl. J. Med..

[93] Conroy T., Desseigne F., Ychou M., Bouché O., Guimbaud R., Bécouarn Y., Adenis A., Raoul J.-L., Gourgou-Bourgade S., De La Fouchardière C. (2011). FOLFIRINOX versus gemcitabine for metastatic pancreatic cancer. N. Engl. J. Med..

[94] Chen J., Hua Q., Wang H., Zhang D., Zhao L., Yu D., Pi G., Zhang T., Lin Z. (2021). Meta-analysis and indirect treatment comparison of modified FOLFIRINOX and gemcitabine plus nab-paclitaxel as first-line chemotherapy in advanced pancreatic cancer. BMC Cancer.

[95] Rapposelli I.G., Casadei-Gardini A., Vivaldi C., Bartolini G., Bernardini L., Passardi A., Frassineti G.L., Massa V., Cucchetti A. (2021). Equivalent Efficacy but Different Safety Profiles of Gemcitabine Plus Nab-Paclitaxel and FOLFIRINOX in Metastatic Pancreatic Cancer. Biomolecules.

[96] Merza N., Farooqui S.K., Dar S.H., Varughese T., Awan R.U., Qureshi L., Ansari S.A., Qureshi H., Mcilvaine J., Vohra I. (2023). Folfirinox vs. Gemcitabine + Nab-Paclitaxel as the First-Line Treatment for Pancreatic Cancer: A Systematic Review and Meta-Analysis. World J. Oncol..

[97] A Wainberg Z., Melisi D., Macarulla T., Cid R.P., Chandana S.R., De La Fouchardière C., Dean A., Kiss I., Lee W.J., O Goetze T. (2023). NALIRIFOX versus nab-paclitaxel and gemcitabine in treatment-naive patients with metastatic pancreatic ductal adenocarcinoma (NAPOLI 3): A randomised, open-label, phase 3 trial. Lancet.

[98] Kolbeinsson H.M., Chandana S., Wright G.P., Chung M. (2023). Pancreatic Cancer: A Review of Current Treatment and Novel Therapies. J. Invest. Surg..

[99] Yamai T., Ikezawa K., Sugimoto N., Urabe M., Kai Y., Takada R., Nakabori T., Uehara H., Kawamura T., Kunimasa K. (2023). Utility of Comprehensive Genomic Profiling Tests for Patients with Incurable Pancreatic Cancer in Clinical Practice. Cancers.

[100] Bekaii-Saab T.S., Yaeger R., Spira A.I., Pelster M.S., Sabari J.K., Hafez N., Barve M., Velastegui K., Yan X., Shetty A. (2023). Adagrasib in Advanced Solid Tumors Harboring a KRAS(G12C) Mutation. J. Clin. Oncol..

[101] Strickler J.H., Satake H., George T.J., Yaeger R., Hollebecque A., Garrido-Laguna I., Schuler M., Burns T.F., Coveler A.L., Falchook G.S. (2023). Sotorasib in KRAS p.G12C-Mutated Advanced Pancreatic Cancer. N. Engl. J. Med..

[102] Esposito I., Kleeff J., Bergmann F., Reiser C., Herpel E., Friess H., Schirmacher P., Büchler M.W. (2008). Most pancreatic cancer resections are R1 resections. Ann. Surg. Oncol..

[103] Verbeke C.S., Leitch D., Menon K.V., McMahon M.J., Guillou P.J., Anthoney A. (2006). Redefining the R1 resection in pancreatic cancer. Br. J. Surg..

[104] Peters N.A., Javed A.A., Cameron J.L., Makary M.A., Hirose K., Pawlik T.M., He J., Wolfgang C.L., Weiss M.J. (2016). Modified Appleby Procedure for Pancreatic Adenocarcinoma: Does Improved Neoadjuvant Therapy Warrant Such an Aggressive Approach?. Ann. Surg. Oncol..

